# The impact of porcine spray-dried plasma protein and dried egg protein harvested from hyper-immunized hens, provided in the presence or absence of subtherapeutic levels of antibiotics in the feed, on growth and indicators of intestinal function and physiology of nursery pigs

**DOI:** 10.1093/tas/txaa095

**Published:** 2020-06-26

**Authors:** Leigh A Ruckman, Amy L Petry, Stacie A Gould, John F Patience

**Affiliations:** Department of Animal Science, Iowa State University, Ames, IA

**Keywords:** functional protein, IgY, in-feed antibiotics, intestinal inflammation and morphology, spray-dried plasma protein, weaned pig

## Abstract

The objective of this experiment was to compare the effects of spray-dried plasma protein (SDPP) and dried egg protein (DEP), without (AB−) or with (AB+) in-feed antibiotics, on growth performance and markers of intestinal health in nursery pigs raised in commercial conditions. This 42-d experiment utilized 1,230 pigs (4.93 ± 0.04 kg body weight; approximately 15–18 d of age). Pigs were randomly assigned to one of six dietary treatments that were arranged as a 2 × 3 factorial of in-feed antibiotics (AB− vs. AB+) and a specialty protein additive (none [CON], porcine SDPP, or DEP). Diets were fed in four phases with phases 3 and 4 as a common diet across all treatments. Specialty protein additives were fed in phases 1 (0–13 d; 3% SDPP, and 0.20% DEP) and 2 (13–26 d; 2% SDPP, and 0.10% DEP). Antibiotics were fed in phases 1–3 (662 mg chlortetracycline [CTC]/kg, 28 mg carbadox/kg, and 441 mg CTC/kg, respectively). Ileal tissue and blood samples were collected from 48 pigs (8 per treatment) on d 20. Data were analyzed using PROC MIXED of SAS (9.4) with pen as the experimental unit; protein additives, antibiotics, and their interaction were fixed effects and block was a random effect. The pigs experienced naturally occurring health challenges in weeks 2 and 4. In the AB− diets, SDPP and DEP increased average daily gain (ADG; *P* = 0.036) and average daily feed intake (ADFI; *P* = 0.040) compared to CON; in the AB+ diets, neither SDPP nor DEP increased ADG or ADFI compared to CON but SDPP did increase these parameters over DEP. The SDPP and DEP diets decreased the number of individual medical treatments compared to CON (*P* = 0.001). The AB+ increased ileal mucosal interleukin (IL)-1 receptor antagonist (*P* = 0.017). Feeding DEP reduced the concentration of mucosal IL-1β compared to CON, but not SDPP (*P* = 0.022). There was a trend for SDPP and DEP to increase villus height:crypt depth compared to CON (*P* = 0.066). Neither antibiotics or protein additive affected serum malondialdehyde concentration or ileal mRNA abundance of *claudin-3* or *4*, *occludin*, or *zonula occludens-1* (*P* > 0.10). In conclusion, SDPP and DEP improved growth performance of weaned pigs in the absence of antibiotics but neither improved growth compared to CON when feeding standard antibiotic levels. The specialty proteins had a positive effect on health; specialty proteins and antibiotics were able to modulate some markers of intestinal inflammation and morphology.

## INTRODUCTION

The weaning process exposes pigs to a multitude of stressors such as dietary and environmental changes, social stress, and an unpredictable array of pathogens. The combination of these stressors typically results in reduced growth rate and feed intake as well as impaired function and integrity of the gut (Lallès et al., 2004; Pluske, 2013; Li et al., 2019). Further, the immune system of a weaned pig is still undergoing development, increasing their susceptibility to enteric pathogens that can cause diarrhea or other gastrointestinal tract (GIT) disorders (Lallès et al., 2007).

In order to combat these performance and health issues, and to reduce mortality and morbidity during the post-weaning period, antibiotics have been used at sub-therapeutic and therapeutic levels in the feed for over five decades (Patience, 2019). However, growing concerns about antimicrobial resistance to antibiotics, consumer demands, and government regulation of antibiotics in livestock diets have prompted the pork industry to seek dietary methods to reduce or eliminate antibiotic use during the nursery stage (Olsen et al., 2018).

Spray-dried plasma protein (SDPP), either from a porcine or bovine source, has been used in nursery diets since the late 1980s and been shown to improve performance and reduce diarrhea in weaned pigs (Peace et al., 2011; Tran et al., 2014). It has been hypothesized that these improvements are the result of increased feed intake, the protective effects of the constituent immunoglobulin rich fraction, and modulation of the immune response and gut barrier structure (Pierce et al., 2005; Peace et al., 2011). This proposed mode of action, as well as reports that SDPP improves performance of unhealthy and/or environmentally challenged pigs, suggests that SDPP could be used to limit or reduce antibiotics in nursery diets (Torrallardona et al., 2002). However, the cost of this ingredient and a desire by some producers to reduce animal products in their feed have prompted the industry to look for alternatives to SDPP (Patterson et al., 2010; Gerber et al., 2014).

Dried egg protein (DEP), specifically egg-yolk antibodies, has garnered attention recently as a promising SDPP alternative. It has been used to protect weaned pigs against diarrhea and enteric diseases since the early 1990s (Wiedemann et al., 1991). This product is produced by drying eggs harvested from hens that are hyper-immunized against specific bacterial antigens known to challenge young pigs (Schade et al., 2005; Li et al., 2015). The resulting ingredient is a concentrated source of egg-yolk immunoglobulin proteins (IgY) that could aid in immune modulation, reduce diarrhea, and improve performance of weaned pigs (Pettigrew, 2006). Supplementing IgY has improved growth performance in enterotoxigenic *Escherichia coli* (ETEC) challenge models (Owusu-Asiedu, Nyachoti, Baidoo et al., 2003; Pozzebon da Rosa et al., 2015). However, the results have been inconsistent in non-challenge studies (Heo et al., 2015; Torrallardona and Polo, 2016). The pork industry needs to have a better understanding of both the impact of IgY under commercial nursery conditions and their mode of action to determine if it can be a practical alternative to SDPP.

Therefore, the objective of this experiment was to compare under commercial conditions the effects of including SDPP or DEP in the diet, with or without subtherapeutic levels of antibiotics in the phase 1 and 2 nursery diets, on growth performance and markers of intestinal physiology and function. It was hypothesized that the SDPP and DEP would improve pig performance in the reduced antibiotic diets, and that this improvement could be mediated by changes in gut integrity and structure, oxidative status, and gut inflammation.

## MATERIALS AND METHODS

All experimental procedures employed in this experiment adhered to principles for the ethical and humane use of animals for research according to the Guide for the Care and Use of Agricultural Animals in Research and Teaching (FASS, 2010) and were approved by the Iowa State University Institutional Animal Care and Use Committee (S-18-129).

### Animals, Housing, and Management

This 42-d experiment was conducted in June and July 2018 in one room of a commercial wean-to-finish research barn located in central Iowa. A total of 1,230 crossbred pigs (PIC 359 × PIC 1050; PIC, Hendersonville, TN) were weaned at 15–18 d of age with a mean body weight (BW) of 4.93 ± 0.04 kg. At weaning, the pigs were vaccinated for porcine circovirus type 2 and *Mycoplasma hyopneumoniae* (Circumvent PCV-MG2, Merck Animal Health, Madison, NJ). Sixty pens were utilized for a total of 10 replicates per dietary treatment. The barn was blocked by location into 10 blocks to balance any potential effect due to position within the barn; dietary treatments were randomly assigned within each block. Each pen housed 20 or 21 mixed sex pigs; both sex and the number of pigs per pen were equalized within block.

The barn was tunnel ventilated and each pen was equipped with a 4-space dry self-feeder, dish waterer, and fully slatted concrete floors. Pigs had ad libitum access to feed and water for the entirety of the experiment. Treatment diets were delivered quantitatively to individual pens using an automatic feed delivery system designed specifically for research purposes (Big Dutchman, Holland, MI).

### Experimental Treatments and Design

Experimental treatments were offered during phase 1 and 2 of the nursery feed budget (Tables 1 and 2) and were arranged as a 2 × 3 factorial comparing in-feed antibiotics (AB; none [AB−] vs. standard [AB+] levels) and a specialty protein additive (ADD; none [CON], porcine SDPP, or DEP). The antibiotics were included in the diets at the expense of corn; phase 1 included 662 mg of chlortetracycline (CTC)/kg (Aureomycin 100, Zoetis, Florham Park, NJ) and phase 2 contained 28 mg of carbadox/kg (Mecadox 10, Phibro Animal Health Corporation, Teaneck, NJ). Due to the health challenges experienced by the pigs, the phase 3 diets contained 441 mg of CTC/kg (Chlormax 50, Zoetis, Florham Park, NJ); all medications utilized in these diets reflected the farm’s veterinary feed directive (VFD) and provided under the direction of the herd health veterinarian, or were added according to the manufacturer’s instructions when no VFD was required. The phase 3 and 4 diets they did not contain the specialty proteins as they were common diets provided to all pigs (Table 3). Phase 4 contained no antibiotics nor specialty proteins. In the DEP diet, Globimax JS (EW Nutrition, Des Moines, IA) was added to CON at the expense of corn at 0.20% in phase 1 and 0.10% in phase 2. The SDPP (AP 920, APC Inc., Ankeny, IA) was added to the diet at 3.0% in phase 1 and 2.0% in phase 2. During diet formulation, no nutritive value was assigned to DEP due to its very low inclusion level; SDPP was formulated into the experimental diets according to its nutrient profile as provided by the manufacturer. Phase 3 and 4 were common diets fed across all treatment groups. Phase 1 and 2 were fed in pelleted form based on the feed budget used by the farm: 2.3 kg of phase 1 (d 0–13) and 5.5 kg of phase 2 (d 13–26). Phase 3 was fed from d 26 to 40 and phase 4 was fed from d 40 to 42, both in mash form.

**Table 1.** Ingredient and nutrient composition of experimental diets (as-fed basis): phase 1^*a*,*b*^

	AB−			AB+		
Item	CON	SDPP	DEP	CON	SDPP	DEP
Ingredient composition, %						
Corn	30.98	33.94	30.78	30.68	33.64	30.48
Oat groats	15.00	15.00	15.00	15.00	15.00	15.00
Soybean meal	20.00	14.21	20.00	20.00	14.21	20.00
Whey permeate	15.00	15.00	15.00	15.00	15.00	15.00
Dried yeast 1^*c*^	5.00	5.00	5.00	5.00	5.00	5.00
Dried yeast 2^*d*^	3.08	3.08	3.08	3.08	3.08	3.08
Fish meal, menhaden	5.00	5.00	5.00	5.00	5.00	5.00
SDPP^*e*^	–	3.00	–	–	3.00	–
DEP^*f*^	–	–	0.20	–	–	0.20
Choice white grease	3.00	3.00	3.00	3.00	3.00	3.00
L-lysine HCl^*g*^	0.50	0.45	0.50	0.50	0.45	0.50
MHA^*h*^	0.23	0.19	0.23	0.23	0.19	0.23
L-threonine	0.15	0.11	0.15	0.15	0.11	0.15
L-tryptophan	0.03	0.03	0.03	0.03	0.03	0.03
Monocalcium phosphate 21%	0.46	0.35	0.46	0.46	0.35	0.46
Limestone	0.82	0.90	0.82	0.82	0.90	0.82
Salt	0.45	0.45	0.45	0.45	0.45	0.45
Nursery VTM premix^*i*^	0.18	0.18	0.18	0.18	0.18	0.18
Choline chloride 60%	0.12	0.12	0.12	0.12	0.12	0.12
CTC^*j*^	–	–	–	0.30	0.30	0.30
Calculated nutrients						
SID Lys^*k*^, %	1.50	1.50	1.50	1.50	1.50	1.50
SID TSAA:Lys^*l*^	0.55	0.55	0.55	0.55	0.55	0.55
SID Thr:Lys	0.60	0.60	0.60	0.60	0.60	0.60
SID Trp:Lys	0.18	0.18	0.18	0.18	0.18	0.18
Ca, %	0.85	0.85	0.85	0.85	0.85	0.85
STTD P^*m*^, %	0.45	0.45	0.45	0.45	0.45	0.45
ME^*n*^, Mcal/kg	3.48	3.51	3.48	3.48	3.51	3.48
NE^*o*^, Mcal/kg	2.35	2.38	2.35	2.35	2.38	2.35
Analyzed nutrients						
Dry matter, %	88.91	89.48	89.41	89.14	89.49	89.30
Ash, %	6.08	5.90	6.19	6.04	6.08	5.91
Crude protein, %	22.41	22.34	23.20	22.70	22.41	22.95
aEE^*p*^, %	7.22	6.88	7.24	7.37	6.92	7.38
SID Lys, %	1.47	1.45	1.44	1.46	1.41	1.45
SID TSAA, %	0.80	0.79	0.81	0.81	0.79	0.83
SID TSAA:Lys	0.54	0.54	0.56	0.55	0.56	0.57
SID Thr, %	0.88	0.86	0.84	0.90	0.86	0.89
SID Thr:Lys	0.60	0.59	0.58	0.62	0.61	0.61
SID Trp, %	0.24	0.26	0.26	0.25	0.28	0.25
SID Trp:Lys	0.16	0.18	0.18	0.17	0.20	0.17
SID Ile, %	0.86	0.80	0.84	0.87	0.79	0.85
SID Ile:Lys	0.59	0.55	0.58	0.60	0.56	0.59
SID Val, %	0.98	0.99	0.95	0.99	0.96	0.97
SID Val:Lys	0.67	0.68	0.66	0.68	0.68	0.67

^*a*^Phase 1 was fed from approximately d 0–13. The feed budget was 2.27 kg/pig.

^*b*^AB, in-feed antibiotics (none [AB−] vs. standard [AB+] levels); ADD, specialty protein additive (none [CON], SDPP, or DEP).

^*c*^Dried yeast 1 is Proplex DY (Archer Daniels Midland Company, Decatur, IL).

^*d*^Dried yeast 2 is Proplex T (Archer Daniels Midland Company, Decatur, IL).

^*e*^SDPP is AP 920 (APC Inc., Ankeny, IA).

^*f*^DEP is Globimax JS (EW Nutrition, Des Moines, IA).

^*g*^L-lysine HCl, L-lysine hydrochloride.

^*h*^MHA, methionine hydroxy analogue.

^*i*^The vitamin and trace mineral (VTM) premix provided per kg of complete diet: 0.21 ppm Cr as Cr_2_O_3_, 10 ppm Cu as CuSO_4_, and Cu-MHA chelate, 0.31 ppm I as calcium iodate, 82 ppm Fe as FeSO_4_, 21 ppm Mn as MnO and Mn-MHA chelate, 0.31 ppm Se as selenium yeast, 170 ppm Zn as ZnO and Zn-MHA chelate, 1,701 IU vitamin D3, 11,337 IU vitamin A, 45.3 IU vitamin E, 4.53 mg menadione, 0.23 mg biotin, 1.7 mg folic acid, 51 mg niacin, 15.6 mg pyridoxine, 28.3 mg pantothenic acid, 8.5 mg riboflavin, 39.7 mg vitamin B12, 514.4 FTU phytase (AxtraPhy, Danisco Animal Nutrition, Marlborough, UK). Premix also contained per kg of complete diet 0.06 g of *bacillus*-based direct-fed-microbial (1.6 × 10^3^ CFU/g).

^*j*^CTC (Aureomycin 100, Zoetis, Florham Park, NJ) was added to the diet at 662 mg/kg.

^*k*^SID, standard ileal digestible.

^*l*^TSAA, total sulfur amino acids (Met + Cys).

^*m*^STTD, standardized total tract digestible.

^*n*^ME, metabolizable energy.

^*o*^NE, net energy.

^*p*^aEE, acid-hydrolyzed ether extract.

**Table 2.** Ingredient and nutrient composition of experimental diets (as-fed basis): phase 2^*a*,*b*^

	AB−			AB+		
Item	CON	SDPP	DEP	CON	SDPP	DEP
Ingredient composition, %						
Corn	51.43	52.70	51.33	51.31	52.58	51.21
Oat groats	5.00	5.00	5.00	5.00	5.00	5.00
Soybean meal	25.00	21.86	25.00	25.00	21.86	25.00
Whey permeate	5.00	5.00	5.00	5.00	5.00	5.00
Dried yeast 1^*c*^	5.00	5.00	5.00	5.00	5.00	5.00
Dried yeast 2^*d*^	1.45	1.45	1.45	1.45	1.45	1.45
SDPP^*e*^	–	2.00	–	–	2.00	–
DEP^*f*^	–	–	0.10	–	–	0.10
Choice white grease	3.00	3.00	3.00	3.00	3.00	3.00
L-lysine HCl^*g*^	0.50	0.44	0.50	0.50	0.44	0.50
MHA^*h*^	0.18	0.16	0.18	0.18	0.16	0.18
L-threonine	0.14	0.11	0.14	0.14	0.11	0.14
L-tryptophan	0.02	0.02	0.02	0.02	0.02	0.02
Monocalcium phosphate 21%	1.05	0.97	1.05	1.05	0.97	1.05
Limestone	1.24	1.30	1.24	1.24	1.30	1.24
Salt	0.70	0.70	0.70	0.70	0.70	0.70
Nursery VTM premix^*i*^	0.16	0.16	0.16	0.16	0.16	0.16
Choline chloride 60%	0.12	0.12	0.12	0.12	0.12	0.12
Carbadox^*j*^	–	–	–	0.125	0.125	0.125
Calculated nutrients						
SID Lys^*k*^, %	1.35	1.35	1.35	1.35	1.35	1.35
SID TSAA:Lys^*l*^	0.55	0.55	0.55	0.55	0.55	0.55
SID Thr:Lys	0.60	0.60	0.60	0.60	0.60	0.60
SID Trp:Lys	0.18	0.18	0.18	0.18	0.18	0.18
Ca, %	0.80	0.80	0.80	0.80	0.80	0.80
STTD P^*m*^, %	0.40	0.40	0.40	0.40	0.40	0.40
ME^*n*^, Mcal/kg	3.41	3.42	3.41	3.41	3.42	3.41
NE^*o*^, Mcal/kg	2.37	2.39	2.37	2.37	2.39	2.37
Analyzed nutrients						
Dry matter, %	88.38	88.53	88.52	88.32	88.47	88.61
Ash, %	5.90	5.40	5.60	5.55	5.81	5.25
Crude protein, %	20.56	21.69	20.99	21.30	21.22	20.90
aEE^*p*^, %	6.44	6.27	6.69	6.07	6.61	6.62
SID Lys, %	1.32	1.39	1.35	1.33	1.33	1.27
SID TSAA, %	0.68	0.75	0.70	0.70	0.74	0.67
SID TSAA:Lys	0.52	0.54	0.52	0.53	0.56	0.53
SID Thr, %	0.76	0.82	0.76	0.75	0.82	0.70
SID Thr:Lys	0.58	0.59	0.56	0.56	0.62	0.55
SID Trp, %	0.23	0.26	0.25	0.25	0.26	0.24
SID Trp:Lys	0.17	0.19	0.19	0.19	0.20	0.19
SID Ile, %	0.78	0.83	0.78	0.80	0.77	0.74
SID Ile:Lys	0.59	0.60	0.58	0.60	0.58	0.58
SID Val, %	0.85	0.96	0.86	0.87	0.89	0.81
SID Val:Lys	0.64	0.69	0.64	0.65	0.67	0.64

^*a*^Phase 2 was fed from approximately d 13–26. The feed budget was 5.45 kg/pig.

^*b*^AB, in-feed antibiotics (none [AB−] vs. standard [AB+] levels); ADD, specialty protein additive (none [CON], SDPP, or DEP).

^*c*^Dried yeast 1 is Proplex DY (Archer Daniels Midland Company, Decatur, IL).

^*d*^Dried yeast 2 is Proplex T (Archer Daniels Midland Company, Decatur, IL).

^*e*^SDPP is AP 920 (APC Inc., Ankeny, IA).

^*f*^DEP is Globimax JS (EW Nutrition, Des Moines, IA).

^*g*^L-lysine HCl, L-lysine hydrochloride.

^*h*^MHA, methionine hydroxy analogue.

^*i*^The VTM premix provided per kg of complete diet: 0.19 ppm Cr as Cr_2_O_3_, 9 ppm Cu as CuSO_4_, and Cu-MHA chelate, 0.28 ppm I as calcium iodate, 73 ppm Fe as FeSO_4_, 19 ppm Mn as MnO and Mn-MHA chelate, 0.28 ppm Se as selenium yeast, 151 ppm Zn as ZnO and Zn-MHA chelate, 1,512 IU vitamin D3, 10,077 IU vitamin A, 40.3 IU vitamin E, 4.03 mg menadione, 0.20 mg biotin, 1.5 mg folic acid, 45 mg niacin, 13.9 mg pyridoxine, 25.2 mg pantothenic acid, 7.6 mg riboflavin, 35.3 mg vitamin B12, 457.2 FTU phytase (AxtraPhy, Danisco Animal Nutrition, Marlborough, UK). Premix also contained per kg of complete diet 0.06 g of *bacillus*-based direct-fed-microbial (1.6 × 10^3^ CFU/g).

^*j*^Carbadox (Mecadox 10, Phibro Animal Health Corporation, Teaneck, NJ) was added in diet at 28 mg/kg.

^*k*^SID, standard ileal digestible.

^*l*^TSAA, total sulfur amino acids (Met + Cys).

^*m*^STTD, standardized total tract digestible.

^*n*^ME, metabolizable energy.

^*o*^NE, net energy.

^*p*^aEE, acid-hydrolyzed ether extract.

**Table 3.** Ingredient and nutrient composition of experimental diets (as-fed basis): phase 3 and 4^*a*^

	Phase 3	Phase 4
Item	CON	CON
Ingredient composition, %		
Corn	50.99	51.39
Corn DDGS^*b*^	15.00	15.00
Soybean meal	28.14	28.14
Choice white grease	2.56	2.56
Lysine sulfate 54.6%	0.69	0.69
DL-methionine	0.14	0.14
L-threonine	0.08	0.08
L-tryptophan	0.04	0.04
Monocalcium phosphate 21%	0.18	0.18
Limestone	1.10	1.10
Salt	0.43	0.43
VTM premix^*c*^	0.15	0.15
Vitamin E (20,000)	0.05	0.05
Copper chloride	0.03	0.03
Phytase^*d*^	0.01	0.01
CTC^*e*^	0.40	-
Calculated nutrients		
SID Lys^*f*^, %	1.26	1.26
SID TSAA:Lys^*g*^	0.58	0.58
SID Thr:Lys	0.62	0.62
SID Trp:Lys	0.20	0.20
Ca, %	0.59	0.59
STTD P^*h*^, %	0.35	0.35
ME^*i*^, Mcal/kg	3.44	3.44
NE^*j*^, Mcal/kg	2.40	2.40
Analyzed nutrients		
Dry matter, %	88.38	88.53
Ash, %	5.90	5.40
Crude protein, %	20.56	21.69
aEE^*k*^, %	6.44	6.27
SID Lys, %	1.34	1.31
SID TSAA, %	0.75	0.67
SID TSAA:Lys	0.56	0.51
SID Thr, %	0.74	0.85
SID Thr:Lys	0.55	0.65
SID Trp, %	0.25	0.22
SID Trp:Lys	0.19	0.17
SID Ile, %	0.75	0.76
SID Ile:Lys	0.56	0.58
SID Val, %	0.83	0.83
SID Val:Lys	0.62	0.63

^*a*^Phase 3 was fed from approximately d 26–40 and phase 4 was fed from d 40–42. Phases were common diets across all treatments.

^*b*^DDGS, distiller’s dried grains with solubles.

^*c*^The VTM premix provided per kg of complete diet: 11,000 IU of vitamin A, 1,650 IU of vitamin D, 33 IU of vitamin E (dl-alpha tocopheryl acetate), 11 IU of vitamin E (d-alpha tocopheryl acetate), 4.4 mg of vitamin K, 0.027 mg of vitamin B_12_, 5.5 mg of riboflavin, 38.5 mg of niacin, 22 mg of pantothenic acid, 0.22 mg of biotin, 1.10 mg of folic acid, 0.88 mg of pyridoxine, 0.395 mg of Co as CoCO_3_, 0.016 g of Cu as CuO or CuSO_4_, 0.22 mg of I as ethylenediamine dihydroiodide (EDDI) or CaI_2_, 0.15 g of Fe as FeSO_4_, 0.03 g of Mn as MnO or MnSO_4_, 0.3 mg of organic Se as selenium yeast, and 0.15 g of Zn as ZnO or ZnSO_4_.

^*d*^Phytase (Optiphos 2000, Huvepharma, Sofia, Bulgaria) included in the diet to provide 250 FTU/kg.

^*e*^CTC (Chlormax 50, Zoetis, Florham Park, NJ) added in diet at 441 mg/kg.

^*f*^SID, standard ileal digestible.

^*g*^TSAA, total sulfur amino acids (Met + Cys).

^*h*^STTD, standardized total tract digestible.

^*i*^ME, metabolizable energy.

^*j*^NE, net energy.

^*k*^aEE, acid-hydrolyzed ether extract.

All diets in this study were formulated to meet or exceed nutrient requirements of the pigs (NRC, 2012). The CON diets contained 20% soybean meal (SBM) in phase 1, 25% in phase 2, and 28% in phases 3 and 4; the DEP diets contained the same inclusion of SBM as CON in phase 1 and 2. The SDPP diets were formulated to contain less SBM than CON and contained only 14.2% and 21.9% SBM in phases 1 and 2, respectively. All diets were formulated to contain the same levels of standardized ileal digestible (SID) lysine, regardless of protein source (phase 1: 1.50% SID lysine; phase 2: 1.35% SID lysine; phase 3 and 4: 1.26% SID lysine). Other potentially limiting amino acids were formulated according to target SID amino acid to SID lysine ratios (NRC, 2012). To the greatest extent possible, all basal ingredients, other than SBM and the experimental ingredients, were included in the diets at the same levels across treatments to minimize the risk of confounding the experimental outcomes. The DEP was weighed out on an analytical scale and delivered to the commercial mill prior to diet manufacturing.

### Medical Treatments and Health Status Characterization

Pigs were individually treated with ceftiofur (Excede, Zoetis, Florham Park, NJ) or enrofloxacin (Enroflox 100, Norbrook Laboratories, Newry, Northern Ireland) as indicated by clinical observation. Pigs that did not respond to medical treatment were removed from the study. During the experiment, individual medical treatments were recorded by product, pen, day, and dosage. The pen, date, BW at removal, and cause were recorded for all mortalities and removals.

Under the direction of a veterinarian and in response to observed diarrhea, lethargy, and respiratory symptoms, medication was also delivered through the water as required: gentamicin sulfate (Gen-Gard, Agrilabs, St. Joseph, MO; 13.2 mg gentamicin sulfate/L of water; d 4–7 and d 11–14), electrolytes (Blue 2, TechMix LLC, Stewart, MN; 7.8 mL of stock solution/L of water; d 14–18 and d 25–28), aspirin (AniPrin LQ-PM, AniMed, Winchester, KY; 7.8 mL of 12% aspirin solution/L of water; d 32–36), and penicillin (Penicillin G Potassium USP, Quo Vademus LLC, Kenansville, NC; 396,258 units of penicillin G/L of water; d 36–39).

Diagnostic necropsies were performed on two pigs on d 11 to confirm exposure to specific pathogens (Table 4). Oral fluids were collected on d 32 from three pens per treatment according to Prickett et al. (2008) to characterize the pathogens present in the barn (Olsen et al., 2018). All diagnostic tests, including diagnostic necropsies, were conducted at the Veterinary Diagnostics Lab (Iowa State University, Ames, IA). If a tissue or oral fluid sample was positive for a specific pathogen, the entire barn was considered to have exposure to that pathogen.

**Table 4.** Results of diagnostic testing throughout experiment (d 0–42)

Day^*a*^	Pathogen^*b*^	Result^*c*^	Testing method^*d*^
11	PEDV	Negative	Fecal PCR
11	PDCoV	Negative	Fecal PCR
11	TGEV	Negative	Fecal PCR
11	Porcine Rotavirus^*e*^	Positive	Fecal PCR
11	*Salmonella* ^*f*^	Positive	Intestinal culture
32	PRRSV^*g*^	Positive	Oral fluid PCR
32	IAV^*h*^	Positive	Oral fluid PCR

^*a*^Day of sample collection.

^*b*^PEDV, porcine epidemic diarrhea virus; PDCoV, porcine deltacoronavirus; TGEV, transmissible gastroenteritis virus; PRRSV, porcine reproductive and respiratory syndrome virus; IAV, influenza A virus.

^*c*^On d 11, 2 pigs showing symptoms of diarrhea, lethargy and gauntness were selected for necropsy. On d 32, oral fluids were collected and tested from 18 pens, spaced equidistantly the barn. If a sample was positive for a specific pathogen, the whole barn was considered to have exposure to that pathogen.

^*d*^PCR, polymerase chain reaction.

^*e*^Pigs were positive for Rotavirus group A, B, and C.

^*f*^ *Salmonella* species were not identified.

^*g*^PRRSV strain was wild type 1-7-4 ORF5.

^*h*^Pigs were positive for influenza H3 and N2.

### Data and Sample Collection

Pigs were weighed by pen at the beginning of the experiment, and at the end of the three weigh periods (d 13, 26, and 42) to determine average daily gain (ADG). Feed intake was recorded for the same periods to determine average daily feed intake (ADFI) and to calculate gain:feed (G:F). Weights and removal dates of pigs were recorded and ADG and ADFI were calculated according to pig days on test.

On d 20, eight pigs per treatment were randomly selected for necropsy from the eight heaviest pens on each treatment (using d 13 pen weight) to maximize the uniformity of necropsied pigs. Prior to euthanasia, blood was collected by jugular venipuncture into a 10 mL vacutainer tube (Becton Dickinson, Franklin Lakes, NJ). Blood samples were placed on ice and allowed to clot prior to centrifugation at 2,000 × *g* for 15 min at 4 °C. The resulting serum was stored at −80 °C for later analysis. The pigs were then euthanized by captive bolt stunning followed by exsanguination. Ileal tissue was collected 10 cm proximal to the ileocecal junction, rinsed with phosphate buffered solution (PBS), snap-frozen in liquid nitrogen, and stored at −80 °C for later analysis. Ileal mucosal scrapings were collected, snap-frozen in liquid nitrogen, and stored at −80 °C. A segment of mid-ileum (approximately 60 cm proximal to the ileal-cecal junction) was collected and fixed in 10% neutral buffered formalin.

### Diet Sample Analysis

Feed samples were taken directly from five feeders per dietary treatment at the end of each phase, pooled within phase and treatment, and homogenized before being stored at −20 °C. Diets were ground to 1 mm particle size using a Wiley Mill (Variable Speed Digital ED-5 Wiley Mill; Thomas Scientific, Swedesboro, NJ), dried to a constant weight at 60 °C, and analyzed in duplicate for dry matter (DM; method 930.15; AOAC, 2007), ash (method 942.05; AOAC, 2007), acid-hydrolyzed ether extract (aEE; method 2003.06; AOAC, 2007), and nitrogen (N; method 990.03; AOAC, 2007; TruMac; LECO Corp., St. Joseph, MI). An ethylenediaminetetraacetate sample (9.56% N; determined to have 9.54 ± 0.05% N) was used for standard calibration and crude protein was calculated as N × 6.25. The intra-assay coefficient of variation (CV) for DM, ash, aEE, and N was 0.8%, 1.0%, 4.8%, and 0.9%, respectively. Diet samples were analyzed for total amino acids at the Agricultural Experiment Station Chemical Laboratories (University of Missouri, Columbia, MO). The SID levels of amino acids were calculated using the assayed total amino acid values and the SID coefficient for each ingredient in the formulation (NRC, 2012).

### Oxidative Stress and Inflammatory Measures

Ileal mucosal samples (50 mg) were homogenized in 4.5 mL of PBS buffer, which contained detergent (0.1%; Triton X-100, Fisher Scientific, Fair Lawn, NJ) and a protease inhibitor cocktail (1:100 ratio to PBS; Sigma-Aldrich, St. Louis, MO) before centrifugation at 10,000 × *g* for 15 min at 4 °C. The supernatant was analyzed for cytokines by an external laboratory (Eve Technologies Corporation, Calgary, AB, Canada) using a multiplex assay. The assay included granulocyte-macrophage colony-stimulating factor (GM-CSF), interferon-γ (IFNγ), interleukin (IL)-1α, IL-1β, IL-1 receptor antagonist (IL-1RA), IL-2, IL-4, IL-6, IL-8, IL-10, IL-12, IL-18, and tumor necrosis factor-α (TNF-α). Malondialdehyde (MDA), a marker of oxidative stress, was measured in serum using a thiobarbituric acid reactive substances (TBARS) kit (TBARS Assay Kit, Cayman Chemical Company, Ann Arbor, MI) as previously described (Armstrong and Browne, 1994; Yagi, 1998). The intra-assay CV was 4.9% and the assay sensitivity was 0–50 µM MDA.

### RNA Isolation and Real-Time Quantitative PCR

Total ribonucleic acid (RNA) of ileal tissue was isolated using a commercial kit (RNeasy Plus Mini Kit, Qiagen, Carlsbad, CA) and Qiagen Tissuelyser II (Germantown, MD). The RNA was treated with a deoxyribonuclease enzyme to prevent genomic deoxyribonucleic acid (DNA) contamination (DNA-free DNA removal kit, Invitrogen, Carlsbad, CA). The RNA concentration was quantified using a spectrophotometer (ND-100; NanoDrop Technologies Inc., Rockland, DE) and all samples had 260:280 nm ratios above 1.8. Complimentary DNA (cDNA) was transcribed from 0.8 μg RNA using the QuantiTect Reverse Transcription Kit (Qiagen, Hilden, Germany) and cDNA samples were diluted 10-fold with nuclease-free water.

Real-time quantitative polymerase chain reaction (RT-qPCR) was performed using iQ SYBR Green Supermix (Bio-Rad Laboratories, Inc., Hercules, CA) in triplicate. The gene-specific primers (Table 5) were diluted to 10 µM with nuclease-free water. Each 20 µL reaction included 10 µL of SYBR Green Supermix, 1 µL of each forward and reverse primer, 3 µL of cDNA and 5 µL of nuclease-free water. A no-reverse transcriptase negative control and a pooled cDNA reference sample were included on each plate. The SYBR Green fluorescence was quantified using a RT-qPCR detection system (iQ5; Bio-Rad Laboratories Inc.) and the following cycling conditions: 5-min initial denaturation at 95 °C followed by 40 RT-qPCR cycles (95 °C for 30 s, 55 or 60 °C for 30 s, and 72 °C for 30 s) and a dissociation curve to verify the amplification of a single RT-qPCR product. Optical System Software (iQ5, version 2.0; Bio-Rad Laboratories Inc.) was used to analyze amplification plots and cycle threshold (Ct) values for each reaction were obtained. The messenger RNA (mRNA) abundance was normalized to a reference gene (ribosomal protein-L19) and the pooled sample. The 2-ΔΔCT method (Livak and Schmittgen, 2001) was used to calculate fold change. The intra-assay CV was less than 4.6% for all RT-qPCR analysis.

**Table 5.** Primers used for RT-qPCR

Gene^*a*^	Primer sequence, 5′→3′ ^*b*^	Product size, base pair	GenBank accession	Annealing temperature, °C
*CLDN3*	F: TTGCATCCGAGACCAGTCC	85	NM_001160075	60
	R: AGCTGGGGAGGGTGACA			
*CLDN4*	F: CAACTGCGTGGATGATGAGA	140	NM_001161637	60
	R: CCAGGGGATTGTAGAAGTCG			
*OCLN*	F: TCGTCCAACGGGAAAGTGAA	95	NM_001163647	55
	R: ATCAGTGGAAGTTCCTGAACCA			
*ZO-1*	F: CTCTTGGCTTGCTATTCG	197	XM_003353439	55
	R: AGTCTTCCCTGCTCTTGC			
*RPL19*	F: AACTCCCGTCAGCAGATCC	147	AF_435591	55
	R: AGTACCCTTCCGCTTACCG			

^*a*^ *CLDN3*, *claudin*-*3*; *CLDN4*, *claudin-4*; *OCLN*, *occludin*; *ZO*-*1*, *zonula occludens*-*1*; *RPL19*, *ribosomal protein*-*L19.*

^*b*^F, forward primer; R, reverse primer.

### Intestinal Morphology

Ileal tissues (fixed in 10% neutral buffered formalin) were embedded in paraffin wax, sectioned, stained with hematoxylin and eosin, and mounted on glass slides (Iowa State University Veterinary Diagnostics Lab, Ames, IA). Images of the slides were taken at 10× power using a DP80 Olympus Camera mounted on an OLYMPUS BX 53/43 microscope (Olympus Scientific, Waltham, MA). Eight villi and crypt pairs per ileal sample were measured using OLYMPUS CellSens Dimension 1.16 software. The ratio of villus height to crypt depth was calculated for each pair (V:C ratio).

### Statistical Analysis

Data were analyzed using one of two mixed models. Model 1 assumed that residuals were normally distributed with a compound symmetry (CS) dependent covariance structure [N0,I CSσe2]. This model was used to analyze growth performance data by weigh period.

$$\begin{array}{l} \begin{array}{lll} Model\;1:\;Y_{ijklm}= & & \;\mu +\tau_{i\;\;\;}+\upsilon_{j}+\tau_{i\;}\upsilon_{j}+a_{k}+\rho_{l\;}\\ & & \;+{\;\;\tau }_{i\;}\rho_{l\;\;\;}+\upsilon_{j}\rho_{l\;\;\;}+\tau_{i\;}\upsilon_{j}\rho_{l\;\;\;}+e_{ijklm}, \end{array} \end{array}$$

where Y_ijklm_ is the observed value for the *m*th experimental unit (pen) within the *l*th period in the *k*th block within the *j*th level of protein additive and *i*th level of antibiotic; *µ* is the general mean; τ_i_ is the fixed effect of the *i*th antibiotic (*i* = AB−, AB+); υ_j_ is the fixed effect of *j*th protein additive (*j* = CON, SDPP, DEP); τ_i_ υ_j_ is the interaction term of antibiotic × additive; a_k_ is the random effect of the *k*th block (*k* = 1–10); ρ_l_ is the fixed effect of period (*l* = 1–3); τ_i_ ρ_l_ is the interaction term of antibiotic × period; υ_j_ρ_l_ is the interaction term of additive × period; τ_i_ υ_j_ρ_l_ is the interaction term of antibiotic × additive × period; and e_ijklm_ is the associated variance as described by the model for Y_ijklm_ (*m* = 1 through 60), assuming a_l_ ~N(0, I σa2), and e_ijklm_ ~N(0,I CSσe2), where *I* is the identity matrix.

Model 2 assumed that residuals were independent and normally distributed [N0, I σe2]. The following mixed model was used to analyze all data except for growth performance by weigh period.

$$\begin{array}{l} Model\;\;2:\;Y_{ijkl}=\;\;\;\mu +\tau_{i}+\upsilon_{j}+\tau_{i}\upsilon_{j}+a_{k}+e_{ijkl} \end{array}$$

where Y_ijkl_ is the observed value for the *l*th experimental unit (pen) within the *k*th block in the *j*th level of protein additive and *i*th level of antibiotic; *μ* is the general mean; τ_i_ is the fixed effect of the *i*th antibiotic (*i* = AB-, AB+); υ_j_ is the fixed effect of *j*th additive (j = CON, SDPP, DEP); τ_i_ υ_j_ is the interaction term of antibiotic × additive; a_k_ is the random effect of the *k*th block (*l* = 1–10); and e_ijkl_ is the associated variance as described by the model for Y_ijkl_ (*l* = 1 through 60), assuming a_k_ ~N(0, Iσa2) and e_ijkl_ ~N(0,I σe2), where *I* is the identity matrix.

Normality and homogeneity of the studentized residuals were verified using the UNIVARIATE procedure of SAS 9.4 (SAS Inst., Cary, NC). Statistical outliers, defined as occurring greater than three standard deviations from the mean, were identified and removed from the analysis. All data and models were analyzed using the MIXED procedure. The CS covariance structure was selected as the best fit for model 1 according to Bayesian Information Criterion for all dependent variables. Fisher’s Least Significant Difference test was used to separate least squares means and differences were considered significant if *P* < 0.05 and trends if 0.05 ≥ *P* < 0.10.

## RESULTS

### Animal Health

The pigs experienced multiple health challenges throughout the experimental period (Table 4). During the second week, two pigs were submitted for necropsy and diagnosed with porcine rotavirus (groups A, B, and C) and *Salmonella* (species not identified). In week 4, analysis of oral fluids confirmed the presence of porcine reproductive and respiratory syndrome virus (PRRSV; wild type 1-7-4 ORF5) and influenza A virus (IAV; H3 and N2 strains). Overall, mortality was 2.0% and morbidity (pigs removed due to illness or injury) was 6.3%. Therefore, the total removal rate was 8.3%.

There was no AB × ADD interaction for the number of medical treatments or total removals (*P* > 0.10; Table 6). The AB levels did not impact the number of medical treatments (*P* > 0.10). The inclusion of SDPP and DEP reduced the number of medical treatments compared to CON (*P* = 0.001). Neither AB nor ADD impacted the number of total removals (*P* > 0.10).

**Table 6.** The effects of in-feed antibiotics and specialty protein additives on medical treatments and removals^*a*,*b*,*c*^

	AB		ADD			Pooled	*P*-value^*d*^	
Item	AB−	AB+	CON	SDPP	DEP	SEM	AB	ADD
Medical treatments, proportion^*e*^	0.856	0.776	0.979^a^	0.735^b^	0.734^b^	0.080	0.157	0.001
Removals, proportion^*f*^	0.088	0.076	0.078	0.080	0.088	0.016	0.396	0.832

^*a*^Data are least square means; *n* = 10 pens per treatment with 20 or 21 pigs per pen, totaling 1,230 pigs; 42 d growth experiment.

^*b*^AB, in-feed antibiotics (none [AB−] vs. standard [AB+] levels); ADD, specialty protein additive (none [CON], SDPP, or DEP).

^*c*^Means within a row without a common superscript (a−b) differ significantly (*P* < 0.05).

^*d*^The AB × ADD interaction was tested but was not significant for either variable (*P* > 0.10).

^*e*^Medical treatments were calculated as the total number of medical treatments administered per pen divided by number of pigs allotted to pen.

^*f*^Removals were calculated as the total number of pigs removed from study (found dead or removed for illness or injury) divided by number of pigs allotted to pen.

### Growth Performance

Overall, AB+ increased ADG (*P* = 0.020; Table 7) and ADFI (*P* = 0.002) in comparison to AB−. However, there were no differences for final BW or G:F (*P* > 0.10). Feeding SDPP resulted in the greatest increase in ADG (*P* = 0.044) and ADFI (*P* = 0.026), followed by DEP and then CON; similarly, there was a trend for SDPP to increase final BW compared to CON, but not DEP (*P* = 0.077).

**Table 7.** The effects of in-feed antibiotics and specialty protein additives on overall growth performance and feed efficiency of pigs^*a*,*b*,*c*^

	AB−			AB+			Pooled	*P*-value		
Item^*d*^	CON	SDPP	DEP	CON	SDPP	DEP	SEM	AB	ADD	AB × ADD
Pens per treatment	10	10	10	10	10	10	**–**	**–**	**–**	**–**
Pigs per treatment, initial	205	205	205	205	205	205	**–**	**–**	**–**	**–**
Pigs per treatment, final^*e*^	179	180	177	182	181	180	**–**	**–**	**–**	**–**
Start BW (d 0)	4.93	4.93	4.93	4.93	4.93	4.92	0.015	0.247	0.372	0.262
End BW (d 42)	15.34	16.01	16.19	16.04	16.44	15.80	0.440	0.196	0.077	0.061
ADG, kg	0.237^a^	0.254^bc^	0.257^bc^	0.258^bc^	0.268^b^	0.251^c^	0.010	0.020	0.044	0.036
ADFI, kg	0.360^a^	0.378^b^	0.383^bc^	0.385^bc^	0.398^c^	0.381^b^	0.013	0.002	0.026	0.040
G:F	0.66	0.67	0.67	0.67	0.67	0.66	0.007	0.984	0.429	0.345

^*a*^Data are least square means; n = 10 pens per treatment with 20 or 21 pigs per pen, totaling 1,230 pigs; 42 d growth experiment; growth calculations included pig days to account for morbidity and mortality.

^*b*^AB, in-feed antibiotics (none [AB−] vs. standard [AB+] levels); ADD, specialty protein additive (none [CON], SDPP, or DEP).

^*c*^Means within a row without a common superscript differ significantly (*P* < 0.05).

^*d*^BW, body weight; ADG, average daily gain; ADFI, average daily feed intake; G:F, gain:feed ratio.

^*e*^Number of pigs removed for necropsy during the experiment: AB-CON: 9; AB-SDPP: 8; AB-DEP: 8; AB+CON: 8; AB+SDPP: 8; AB+DEP: 9.

The CON (AB+) diet improved overall ADG and ADFI but not G:F compared to CON (AB−) diets (*P* < 0.05; Table 7). There was an AB × ADD interaction for overall ADG (*P* = 0.036) and ADFI (*P* = 0.040) with a trend for an interaction for final BW (*P* = 0.061). The SDPP and DEP increased ADG, ADFI and final BW compared to CON in the AB− diets, but not in the AB+ diets. In the AB+ diets, SDPP increased ADG and ADFI over DEP.

Considering growth performance by period, the AB × ADD × period interaction was not significant for any growth parameters (*P* > 0.10), therefore only the main effects of AB or ADD are presented (Table 8). The AB+ diet increased G:F compared to AB− in period 1 but did not differ in periods 2 or 3 (*P* = 0.018). Similarly, feeding SDPP increased G:F compared to CON and DEP in period 1, but ADD did not have an impact in subsequent periods (*P* = 0.019).

**Table 8.** The effects of in-feed antibiotics and specialty protein additives on growth performance and feed efficiency of pigs by weigh period analyzed as a mixed model with a time dependent variance structure^*a*,*b*,*c*,*d*^

	AB		ADD					*P*-value^*f*^				
Item^*e*^	AB−	AB+	CON	SDPP	DEP	Pooled SEM	AB	ADD	Period	AB × Period	ADD × Period	AB × ADD
BW, kg						0.20	0.006	0.003	<0.001	0.575	0.642	0.010
d 0	4.93	4.92	4.93	4.93	4.92							
d 13	6.15	6.35	6.17	6.35	6.23							
d 26	9.68	10.00	9.60	10.05	9.87							
d 42	15.85	16.09	15.69	16.23	15.99							
ADG, kg						0.012	0.017	0.022	<0.001	0.670	0.687	0.032
d 0–13	0.095	0.111	0.097	0.111	0.102							
d 13–26	0.274	0.283	0.265	0.289	0.282							
d 26–42	0.369	0.373	0.372	0.374	0.368							
ADFI, kg						0.015	0.002	0.019	<0.001	0.893	0.955	0.051
d 0–13	0.142	0.153	0.144	0.150	0.147							
d 13–26	0.404	0.422	0.403	0.425	0.412							
d 26–42	0.561	0.572	0.556	0.574	0.569							
G:F						0.01	0.088	0.010	<0.001	0.018	0.019	0.073
d 0–13	0.67^x^	0.72^y^	0.67^ab^	0.73^c^	0.69^a^							
d 13–26	0.67^x^	0.67^x^	0.65^b^	0.68^ab^	0.68^ab^							
d 26–42	0.66^x^	0.65^x^	0.68^ab^	0.65^b^	0.65^b^							

^*a*^Data are least square means; n = 10 pens per treatment with 20 or 21 pigs per pen, totaling 1,230 pigs; 42 d growth experiment; growth calculations included pig days to account for morbidity and mortality.

^*b*^AB, in-feed antibiotics (none [AB−] vs. standard [AB+] levels); ADD, specialty protein additive (none [CON], SDPP, or DEP).

^*c*^Weigh periods: period 1 (d 0–13), period 2 (d 13–26), and period 3 (d 26–42).

^*d*^Within a dependent variable, means without a common superscript (x−y or a−c) differ significantly (*P* < 0.05).

^*e*^BW, body weight; ADG, average daily gain; ADFI, average daily feed intake; G:F, gain:feed ratio.

^*f*^The AB × ADD × period interaction was tested but was not significant for any of the variables (*P* > 0.10).

### Oxidative Stress and Inflammatory Measures

The inclusion of AB or ADD did not impact the levels of serum MDA (*P* > 0.10; Table 9). There was no effect of AB or ADD, or their interaction, on the following ileal mucosa cytokines: IFNγ, IL-1α, IL-2, IL-4, IL-6, IL-8, IL-10, or IL-12 (*P* > 0.10). The concentrations of GM-CSF and TNF-α were not detectable in any of the samples. Feeding DEP resulted in the lowest concentration of IL-1β compared to CON, with SDPP being intermediate between them (*P* = 0.022). The concentration of IL-1RA was increased by feeding AB+ compared to AB− (*P* = 0.017). An AB × ADD interaction was observed for IL-18 as the concentration did not differ in the AB− diets but was significantly increased in the CON diet compared to SDPP and DEP when AB+ was fed (*P* = 0.012).

**Table 9.** The effects of in-feed antibiotics and specialty protein additives on oxidative stress and ileal mucosa cytokines^*a*,*b*,*c*^

	AB−			AB+			Pooled	*P*-value		
Item	CON	SDPP	DEP	CON	SDPP	DEP	SEM	AB	ADD	AB × ADD
Malondialdehyde, µM	16.60	14.46	13.45	16.23	16.65	16.09	1.35	0.184	0.481	0.492
Cytokines^*d*^, ng/g										
IFNγ	21.41	21.82	18.91	25.26	23.00	22.82	2.72	0.166	0.624	0.838
IL-1α	5.26	4.56	3.36	4.16	4.96	4.85	0.93	0.705	0.684	0.320
IL-1β	16.97	15.54	11.65	23.16	22.23	11.27	3.45	0.113	0.022	0.473
IL-1ra	10.25	10.25	8.79	15.18	11.75	12.54	1.65	0.017	0.422	0.577
IL-2	1.12	1.25	1.10	1.27	1.12	1.18	0.14	0.757	0.923	0.596
IL-4	1.13	1.24	1.14	1.37	1.09	0.78	0.20	0.559	0.332	0.313
IL-6	0.77	0.86	0.62	1.07	0.83	0.94	0.15	0.111	0.636	0.425
IL-8	256.91	243.68	200.88	271.47	226.15	215.31	31.26	0.881	0.203	0.840
IL-10	0.66	0.72	0.41	0.82	0.65	0.67	0.11	0.194	0.139	0.266
IL-12	7.93	6.72	5.86	7.41	6.78	6.98	1.03	0.784	0.429	0.699
IL-18	38.03^a^	44.92^a^	37.20^a^	124.73^b^	57.23^a^	49.75^a^	15.62	0.002	0.019	0.012

^*a*^Data are least square means; n = 8 replicates per treatment; serum and ileal tissue samples were collected on d 20.

^*b*^AB, in-feed antibiotics (none [AB−] vs. standard [AB+] levels); ADD, specialty protein additive (none [CON], SDPP, or DEP).

^*c*^Means within a row without a common superscript differ significantly (*P* < 0.05).

^*d*^Interferon-γ (IFNγ); interleukin-1α (IL-1α); interleukin-1β (IL-1β); interleukin-1 receptor antagonist (IL-1ra); interleukin-2 (IL-2); interleukin-4 (IL-4); interleukin-6 (IL-6); interleukin-8 (IL-8); interleukin-10 (IL-10); interleukin-12 (IL-12); and interleukin-18 (IL-18).

### Ileal Gene Transcription

The mRNA abundance of *claudin-3* (*CLDN3*), *CLDN4*, *occludin* (*OCLN*), or *zonula-occludens-1* (*ZO-1*) was not impacted by AB or ADD (*P* > 0.10; Table 10). No AB × ADD interactions were observed for the mRNA abundance of these genes (*P* > 0.10).

**Table 10.** The effects of in-feed antibiotics and specialty protein additives on relative ileal gene mRNA abundance^*a*,*b*,*c*^

	AB		ADD			Pooled	*P*-value^*e*^	
Gene^*d*^	AB−	AB+	CON	SDPP	DEP	SEM	AB	ADD
*CLDN3*	1.64	1.38	1.20	2.00	1.33	0.35	0.505	0.187
*CLDN4*	1.60	1.17	1.22	1.37	1.56	0.36	0.267	0.969
*OCLN*	1.38	1.08	1.09	1.34	1.25	0.24	0.477	0.541
*ZO-1*	1.08	0.99	0.93	1.04	1.15	0.16	0.916	0.779

^*a*^Data are least square means; n = 8 replicates per treatment; ileal tissue samples were collected on d 20.

^*b*^AB, in-feed antibiotics (none [AB−] vs. standard [AB+] levels); ADD, specialty protein additive (none [CON], SDPP, or DEP).

^*c*^Means within a row without a common superscript differ significantly (*P* < 0.05).

^*d*^ *CLDN3*, *claudin*-*3*; *CLDN4*, *claudin*-*4*; *OCLN*, *occludin*; *ZO*-*1*, *zonula occludens*-*1*.

^*e*^The AB × ADD interaction was tested but was not significant for either variable (*P* > 0.10).

### Morphology of Gut

There was a trend for DEP to increase villus height compared to CON, with SDPP being intermediate between them (*P* = 0.098; Table 11). In AB− diets, there was a trend for SDPP to result in shallower crypts compared to CON with DEP being intermediate between them; however, in AB+ diets, neither SDPP nor DEP impacted crypt depth compared to CON (*P* = 0.098). There was a trend for feeding SDPP and DEP to result in a larger V:C ratio than CON (*P* = 0.066).

**Table 11.** The effects of in-feed antibiotics and specialty protein additives on ileal morphology^*a*,*b*,*c*^

	AB−			AB+			Pooled	*P*-value		
Item	CON	SDPP	DEP	CON	SDPP	DEP	SEM	AB	ADD	AB × ADD
Villus height, µm	321.9	353.4	371.2	341.1	359.2	355.2	14.92	0.810	0.098	0.499
Crypt depth, µm	270.7	244.3	262.3	258.8	267.0	256.6	10.27	0.802	0.550	0.098
Villi height:crypt depth	1.30	1.56	1.51	1.44	1.48	1.53	0.08	0.700	0.066	0.313

^*a*^Data are least square means; n = 8 replicates per treatment; ileal tissue samples were collected on d 20 and fixed in 10% neutral-buffered formalin before histology slides were made.

^*b*^AB, in-feed antibiotics (none [AB−] vs. standard [AB+] levels); ADD, specialty protein additive (none [CON], SDPP, or DEP).

^*c*^Means within a row without a common superscript differ significantly (*P* < 0.05).

## DISCUSSION

The proposed mode of action for SDPP is that it increases feed intake by improving feed palatability and benefits intestinal health by reducing intestinal permeability and modulating immune responses (Ermer et al., 1994; Peace et al., 2011; Tran et al., 2014). These beneficial effects have been attributed to the immunoglobulin G (IgG)-rich fraction of SDPP (Pierce et al., 2005). The DEP is a concentrated source of IgY, the main circulating antibody in chickens that has a similar biological role as mammalian IgG (Abbas et al., 2019). These immunoglobulins can counteract pathogen activity in the GIT of weaned pigs by inhibiting adhesion to the intestinal epithelium, possibly reducing other symptoms of enteric diseases such as increased intestinal inflammation or permeability (Bosi et al., 2004; Abbas et al., 2019). Therefore, SDPP and DEP could be beneficial to pigs that are overcoming weaning stress and exposure to new pathogens. In this study, we evaluated the impact of SDPP or DEP, with or without in-feed antibiotics, on growth performance and intestinal permeability, inflammation, and morphology of nursery pigs raised under commercial conditions.

When AB− diets were fed, SDPP and DEP increased overall ADG and ADFI and tended to increase final BW compared to the CON diet. Therefore, these specialty proteins could be beneficial to pork producers that are trying to reduce their usage of in-feed antibiotics. Improved performance from feeding SDPP in antibiotic-free studies has been reported in multiple publications, but the benefits of SDPP tend to be more pronounced during the first 2 weeks after weaning (van Dijk et al., 2001; Pérez-Bosque et al., 2016). Further, SDPP has been shown to benefit growth in commercial environments or during pathogen challenges (Coffey and Cromwell, 1995; Bosi et al., 2004). The growth response to feeding DEP or purified IgY to weaned pigs has been more inconsistent in the literature. Torrallardona and Polo (2016) and Crenshaw et al. (2017) reported that DEP (0.2% or 0.44%) did not improve growth in antibiotic-free diets compared to the control diet and was outperformed by diets containing 5% or 6% SDPP. The specificity of the pathogens that a hen was hyperimmunized against can have a large impact on the efficacy of IgY (Li et al., 2015). Studies that have used a disease challenge model, such as ETEC, and have supplemented IgY that is specific to these pathogens, have seen greater growth responses compared to the control diet (Owusu-Asiedu, Nyachoti, Baidoo et al., 2003; Pozzebon da Rosa et al., 2015). However, the pathogen specificity of the DEP used in this trial is unknown.

When AB+ diets were fed, the SDPP and DEP did not have an impact on overall growth performance compared to CON. Therefore, these specialty proteins do not provide as large of a benefit in feeding systems that are using standard antibiotic levels. Bikker et al. (2004) reported that the d 0–14 growth response to SDPP, compared to the control, was greater when feeding antibiotic-free diets rather than antibiotic-positive diets. It was hypothesized that SDPP provided more benefit to pigs fed antibiotic-free diets because pathogenic bacteria were more likely to colonize their GIT compared to pigs fed medicated diets (Bikker et al., 2004). To our knowledge, there are no published studies that have evaluated DEP using a factorial arrangement with antibiotics.

In this study, feeding CON with AB+ compared to AB− increased ADG and ADFI. A proposed mode of action for antibiotics is improved performance by inhibiting bacterial infections in the GIT, which can reduce microbial use of nutrients and abundance of growth-depressing metabolites and toxins (Cromwell, 2002; Gaskins et al., 2006). The growth promoting ability of antibiotics has been well-documented in the literature, but the magnitude of response to antibiotics can be influenced by medication type and dosage or health status of pigs (Cromwell, 2002; Jacela et al., 2009).

During this experiment, the pigs faced multiple naturally-occurring health challenges. Porcine rotavirus and *Salmonella*, both diagnosed on d 11, are associated with watery diarrhea and depressed growth and feed intake (Turner et al., 2002; Corl et al., 2007). On d 32, PRRSV and IAV were diagnosed after severe lethargy and coughing were observed. Despite these severe health challenges, mortality and morbidity were only 2% and 6.3%, respectively. A previous nursery trial that took place in the same facility reported 1.8% mortality and 6.1% morbidity with a naturally occurring PRRSV challenge, so the rates in our study were not abnormal (Olsen et al., 2018). The number of administered medical treatments was quite high, but pigs that were fed SDPP or DEP required 25% fewer medical treatments than those fed CON. To our knowledge, there is no published literature that reports the impact of SDPP or DEP on the number of administered medical treatments.

Pro-inflammatory cytokines play a crucial role in the GIT immune system by recruiting and activating cells to signal an immune response against pathogens (Pié et al., 2004). However, uncontrolled inflammatory responses result in increased intestinal permeability and decreased growth performance, probably due to the partitioning of nutrients and energy away from growth to support the immune response (Huntley et al., 2018). In this study, feeding DEP significantly reduced the mucosal concentration of pro-inflammatory IL-1β compared to CON. The IgY in DEP can block the adhesion of pathogens to the epithelial lining, preventing or reducing the need for an inflammatory response and overproduction of IL-1β (Wang et al., 2019). The mucosal concentration of pro-inflammatory IL-18 was increased by CON when AB+ diets were fed, but the concentration was not affected by the other diets. It has been reported that the mRNA abundance of IL-18 by porcine immune cells is increased during bacterial infections to increase resistance (Foss et al., 2001). However, antibiotics are known to reduce microbial infections and so the increased mucosal IL-18 in pigs fed CON with AB+ diets cannot be explained. Feeding AB+ diets increased the mucosal concentration of anti-inflammatory IL-1RA compared to AB− diets. The IL-1RA can prevent overactivation of the immune response by inhibiting the pro-inflammatory IL-1 family, so this outcome points to an immune modulating effect of antibiotics (Netea et al., 2015).

A physical defense mechanism of the GIT immune system is the intestinal epithelial barrier and mucous layer (Gao et al., 2013; France and Turner, 2017). Paracellular permeability between epithelial cells is maintained by tight junction (TJ) proteins, but the mRNA abundance of these proteins is decreased in weaned pigs (Hu et al., 2013). Disruption of the TJ proteins and increased paracellular permeability can result in the translocation of pathogens or endotoxins from the lumen into the body, which may activate the GIT immune response (Awad et al., 2017). The mRNA abundance of CLDN3, CLDN4, OCLN, or ZO-1 was not affected by antibiotics or protein additive in this study. Zhang et al. (2016) reported that feeding SDPP increased the abundance of ZO-1 and CLDN1 but did not change abundance of OCLN. To the authors knowledge, there are no published studies that report the impact of DEP on the mRNA abundance of TJ proteins. It is known that pro-inflammatory cytokines, such as IFNγ and TNF-α, can disrupt the regulation of TJ proteins (Al-Sadi et al., 2009). Further, these cytokines have been shown to reduce the mRNA abundance of OCLN and ZO-1 (Youakim and Ahdieh, 1999; Mankertz et al., 2000). Therefore, the lack of differences in the mucosal concentration of IFNγ could partially explain why we did not observe altered abundance of TJ proteins (Hu et al., 2013).

The weaning process causes villus atrophy in the small intestine, resulting in less surface area for nutrient digestion and absorption (Montagne et al., 2007). Villus atrophy without crypt hyperplasia is caused by decreased feed intake after weaning as this slows the crypt-cell production rate and reduces the number of new enterocytes that migrate to the villi tip (Pluske et al., 1997). Feeding DEP tended to increase villus length compared to the CON diet, but there was no change in crypt depth. The SDPP numerically increased villus length compared to CON and tended to reduce crypt depth in the AB− diets. Therefore, feeding the SDPP and DEP diets tended to increase the V:C ratio, indicating that these pigs had more absorptive capabilities than those fed the CON diet (Nabuurs et al., 1993). Further, since crypt hyperplasia was not observed in pigs fed the CON diet, it is likely that the villus atrophy occurred due to decreased feed intake. Improvements in the ileal V:C ratio from feeding SDPP or DEP have been reported in other publications (Owusu-Asiedu, Nyachoti, and Marquardt, 2003; Nofrarías et al., 2006).

In conclusion, the SDPP and DEP improved growth rate and feed intake of pigs in AB− diets. When AB+ diets were fed, the SDPP and DEP did not improve growth performance compared to CON. Further, SDPP and DEP reduced the number of required medical treatments. The mucosal cytokine results indicate that specialty proteins and antibiotics can modulate the intestinal immune response of weaned pigs. Feeding specialty proteins had a slight beneficial impact on ileal morphology. These results support our hypothesis that feeding specialty proteins in antibiotic-free diets would improve pig performance, and the modulation of intestinal inflammation and gut morphology could partially explain these improvements. This experiment provides novel data about the impact of specialty proteins on weanling pig performance and intestinal health when differing levels of antibiotics are fed in a commercial setting. Both SDPP and DEP could provide benefit to pork producers that are reducing their dietary antibiotic usage, but further research is still needed to characterize the mode of action of DEP and determine the full extent of its impact on weaned pigs.
